# Comparative Bioassay-Guided Fractionation of *Citrus* Species: Phytochemical Characterization and Nanoformulation of a Polyphenol-Rich Leaf Fraction from *Citrus aurantifolia* for Skin Anti-Aging Applications

**DOI:** 10.3390/nu18132130

**Published:** 2026-07-01

**Authors:** Noha Swilam, Khaled A. Nematallah, Amgad Albohy, Noha M. Badawi, Sameh S. Gad, Maha M. Shouman, Saeed S. Al-Ghamdi, Abdullah R. Alzahrani, Nahla Ayoub

**Affiliations:** 1 Department of Pharmacognosy, Faculty of Pharmacy, The British University in Egypt (BUE), Cairo 11837, Egypt; khaled.nematallah@bue.edu.eg; 2 Department of Medicinal Chemistry, College of Pharmacy, University of Sharjah, Sharjah 27272, United Arab Emirates; aalbohy@sharjah.ac.ae; 3 Department of Pharmaceutics and Pharmaceutical Technology, Faculty of Pharmacy, The British University in Egypt (BUE), Cairo 11837, Egypt; noha.alaa@bue.edu.eg; 4 Department of Pharmacology and Toxicology, Faculty of Pharmacy, October University for Modern Sciences, and Arts MSA, 6th October City 12451, Egypt; sshaban@msa.edu.eg (S.S.G.); mamoussa@msa.edu.eg (M.M.S.); 5 Department of Pharmacology and Toxicology, Faculty of Medicine, Umm Al-Qura University, Makkah 24382, Saudi Arabia; sskghamdi@uqu.edu.sa (S.S.A.-G.); aralzahrani@uqu.edu.sa (A.R.A.)

**Keywords:** *Citrus aurantifolia*, LC–MS/MS, antioxidant activity, anti-aging enzyme, wound-healing, nanovesicles, molecular dynamics

## Abstract

Background: Skin aging is driven by oxidative stress and ultraviolet (UV) exposure, leading to extracellular matrix degradation and loss of skin elasticity. This study aimed to identify the most biologically active *Citrus* species using a bioassay-guided approach and evaluate its potential for dermal applications. Methods: Hydroalcohol extracts and ethyl acetate fractions of *Citrus sinensis*, *Citrus aurantifolia*, and *Citrus reticulata* leaves were screened for antioxidant, enzyme-inhibitory, and polyphenol content. The most active fraction was characterized by UPLC-PDA and LC–MS/MS, formulated into Span-based nanovesicles, and evaluated for physicochemical properties and drug release. Biological activity was assessed using an in vitro scratch wound-healing assay on human dermal fibroblasts and a UVA-induced photoaging mouse model, supported by molecular dynamics simulations. Results: The ethyl acetate fraction of *C. aurantifolia* (CAE) exhibited the highest biological activity among the tested samples. This fraction showed potent antioxidant activity (DPPH IC_50_ = 3.53 ± 0.05 µg/mL), marked inhibition of elastase (91.3%), collagenase (92.0%), and tyrosinase (80.2%), and a high total flavonoid content (110.49 mg rutin equivalents/g). Phytochemical profiling of CAE tentatively identified fourteen compounds, predominantly flavonoids, with hesperidin (30.4 mg/g) as a major constituent. The optimized nanovesicles (184 ± 0.9 nm, PDI 0.10, EE% 75.0%) enabled sustained hesperidin release. CAE and CAEnp enhanced fibroblast migration and accelerated wound closure at 24 h (*p* < 0.05). In vivo, CAEnp improved UVA-induced histopathological alterations and modulated oxidative stress-related markers by reducing p62/SQSTM1 by 28.7%, Keap1 expression to 21% compared with the CAE-treated group, and enhancing Nrf2, ARE, and NQO1 expression by 54.1%, 28.3%, and 57%, respectively. Molecular dynamics simulations supported stable hesperidin binding to elastase and suggested possible modulation of collagenase flexibility. Conclusions: The polyphenol-rich leaf fraction from *C. aurantifolia*, identified through comparative bioassay-guided fractionation, demonstrated antioxidant, enzyme-inhibitory, wound-healing, and photoprotective effects, particularly after nanoformulation. These findings support its potential for further development as a natural topical anti-aging candidate.

## 1. Introduction

Understanding the causes of skin aging is crucial in cosmetic dermatology, as it helps create innovative skincare products that not only fight the visible signs of aging but also improve our overall well-being and boost self-confidence. Skin aging is a complex biological process driven by both intrinsic and extrinsic factors. Intrinsic factors include genetic predisposition and age-related hormonal alterations; whereas, extrinsic influences include ultraviolet (UV) radiation, environmental pollution, and oxidative stress [1]. These environmental factors promote excessive generation of reactive oxygen species (ROS), leading to cellular damage, breakdown of collagen and elastin fibers, and impairment of the skin’s overall structural integrity [2]. Products rich in antioxidants play a key role in preventing and slowing this process by neutralizing ROS and supporting the skin’s natural defense systems [3]. Conversely, many synthetic cosmetic ingredients can cause irritation, sensitization, or allergic reactions, further encouraging the shift toward natural alternatives [4]. Green cosmeceuticals are cosmetic products derived from plants, minerals, and other natural sources and are increasingly recognized for their safety, sustainability, and potential for fewer side effects [5]. The use of botanical extracts in skincare is not new; such preparations have been applied for thousands of years in various traditional medicine systems [6]. In modern formulations, these extracts are valued for their bioactive compounds as flavonoids, polyphenols, and vitamins, which exhibit antioxidant activity, stimulate collagen biosynthesis, and regulate enzymes involved in maintaining the extracellular matrix and skin elasticity [7].

Polyphenolic compounds have attracted considerable interest due to their protective effects against UV-induced skin damage, including photoaging and hyperpigmentation [8]. Flavonoids, as major constituents of the human diet, have many reported beneficial qualities [4]. Within this context, Genus *Citrus* belonging to family Rutaceae, including oranges, lemons, limes, and grapefruits, has emerged as a valuable source of bioactive compounds [4,5]. These fruits were used for a long time in traditional medicine due to their numerous health benefits. Recent research has reported the anti-aging properties of *Citrus* species, notably in their ability to reduce oxidative stress through high levels of antioxidants [9,10]. Additionally, *Citrus* extracts have been reported to enhance collagen synthesis and support skin elasticity, making them a promising natural candidate for anti-aging skincare products [5,11].

The selection of *Citrus* species in the present study was based on their wide cultivation in Egypt, economic importance, and richness in polyphenol and flavonoid constituents with reported antioxidant and skin-protective properties [12]. Among the large number of *Citrus* species, *C. sinensis*, *C. aurantifolia*, and *C. reticulata* were selected because they are commonly cultivated *Citrus* crops in Egypt and generate considerable amounts of leaf biomass during cropping and agricultural handling. Although *Citrus* leaves may provide lower extraction yields compared with fruit peels or other plant parts, their use is highly relevant from a sustainability perspective, as these leaves are often discarded as agricultural by-products. Valorizing this underutilized biomass may therefore contribute to waste reduction while providing a sustainable source of natural bioactive compounds for value-added dermal and cosmeceutical applications. This approach aligns with current sustainability principles in the cosmetic and dermal product industries by supporting waste reduction, resource efficiency, and the development of naturally derived cosmeceutical actives. Accordingly, the present study adopted a comparative bioassay-guided approach to identify the most promising leaf-derived *Citrus* fraction for further phytochemical characterization, nanoformulation, and biological evaluation [13].

While the efficacy of plant-derived antioxidants is well established, their topical delivery remains a challenge due to issues such as poor solubility, instability, and limited skin penetration [14]. Nanotechnology offers innovative solutions to these limitations. Among emerging approaches, Span-based nanoformulations stand out for their combined vesicular and nanoparticulate characteristics [15]. These systems are typically composed of a non-ionic surfactant (often Span) and edge activators such as Tween^®^ 80. Span 60 has demonstrated superior vesicle stability compared to other Spans (e.g., Span 40 or Span 80) [16]. Tween^®^ 80 functions as an edge activator, enhancing vesicle flexibility and facilitating drug penetration by transiently increasing the permeability of biological membranes [16]. The result is improved entrapment efficiency, enhanced stability via steric stabilization, and greater bioavailability of encapsulated actives. Span-based carriers are elastic, biodegradable, and easily modulated, offering distinct advantages over conventional vesicular delivery systems [17].

Despite the well documented bioactivity of *Citrus* species, most studies have focused on fruit peel, juice, or essential oils; whereas, the leaves remain comparatively underexplored despite their potential richness in polyphenol compounds and their value as sustainable, underutilized agricultural by-products. Previous *Citrus*-based cosmeceutical studies have mainly investigated conventional plant parts such as peels and fruits or focused on isolated *Citrus* flavonoids [6,10]. Similarly, several nanoformulation approaches have been reported for plant-derived flavonoids; however, these studies commonly address individual compounds rather than bioassay-guided *Citrus* leaf fractions [4,9,10]. Therefore, the present work is distinct in its focus on *Citrus* leaves as a sustainable source of bioactive compounds and in its integrated evaluation of a selected leaf-derived fraction rather than a single isolated molecule. Moreover, the effective topical application of plant-derived antioxidants is frequently limited by poor stability, low bioavailability, and restricted skin penetration. Few studies have adopted an integrated strategy combining comparative bioassay-guided selection, phytochemical characterization, advanced nanovesicular delivery, and multi-level biological evaluation. Therefore, this study aimed to comparatively investigate the anti-aging potential of hydroalcohol extracts and ethyl acetate fractions from the leaves of three *Citrus* species: sweet orange (*Citrus sinensis*), lime (*Citrus aurantifolia*), and mandarin (*Citrus reticulata*). The most active fraction was selected based on antioxidant activity, inhibition of skin-aging-related enzymes, and polyphenol/flavonoid content. It was then characterized using UPLC-PDA and LC–MS/MS and formulated into a Span-based nanovesicular system to enhance its dermal applicability. The biological potential of the selected fraction and its nanoformulation was further evaluated using human dermal fibroblast assays, a UVA-induced photoaging mouse model, and molecular dynamics simulations. To the best of our knowledge, this is the first integrated study to characterize a polyphenol-rich ethyl acetate fraction from *C. aurantifolia leaves* and evaluate its nanoformulation for skin anti-aging applications.

## 2. Results

### 2.1. Comparative Screening of Citrus Extracts and Fractions

#### 2.1.1. Evaluation of Cytotoxicity in Human Skin Fibroblast

**Figure 1:**
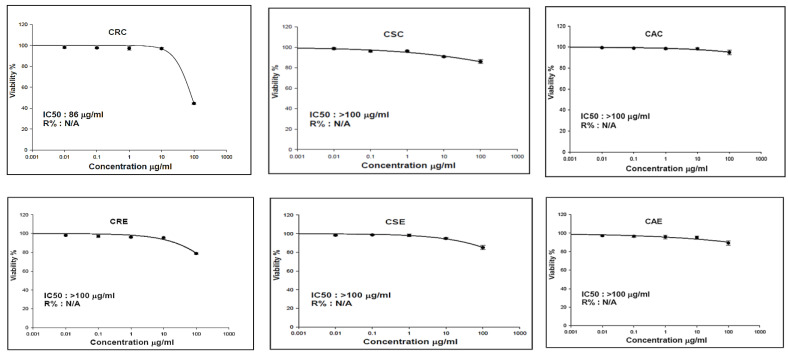
In vitro cytotoxicity of different *Citrus* extracts and fractions in human skin fibroblasts. Cell viability was determined using the SRB-U assay after 24 h treatment with increasing concentrations (0.01–100 µg/mL) of (CRC, CSC, CAC) and (CRE, CSE, CAE). Results are expressed as mean ± SD of three independent biological experiments (*n* = 3), each performed in triplicate.

Cytotoxic effects of the extracts and fractions were assessed in human skin fibroblasts (HSF) using the SRB-U assay. Following 24 h of treatment, most samples did not cause a notable decrease in cell viability over the tested concentration range of 0.01–100 μg/mL (Figure 1). Cytotoxicity was detected only for the CRC crude extract at the maximum concentration tested (100 μg/mL), with an IC_50_ of 86 μg/mL. In contrast, the remaining samples had IC_50_ values above 100 μg/mL, indicating a favorable safety profile within the tested range.

#### 2.1.2. Antioxidant Assays

The antioxidant activity of the hydroalcohol crude extracts (CAC, CSC, CRC) and their corresponding ethyl acetate fractions (CAE, CSE, CRE) were evaluated using DPPH, β-carotene bleaching, and ORAC assays.

The antioxidant activity of the hydroalcohol extracts and ethyl acetate fractions was assessed using DPPH, β-carotene bleaching, and ORAC assays. In the DPPH assay, the hydroalcohol extracts showed moderate radical-scavenging activity, with CAC exhibiting the lowest IC_50_ value among the crude extracts (13.2 ± 0.17 µg/mL), followed by CSC (16.8 ± 0.24 μg/mL) and CRC (20.1 ± 0.30 μg/mL). The ethyl acetate fractions showed stronger activity, with CAE displaying the highest potency (IC_50_ = 3.53 ± 0.05 µg/mL), followed by CSE (5.31 ± 0.11 μg/mL) and CRE (10.5 ± 0.20 μg/mL).

A similar trend was observed in the β-carotene-linoleic acid bleaching assay. Among the crude extracts, CAC again demonstrated the most effective inhibition (IC_50_ = 4.20 ± 0.21 μg/mL), followed by CSC (5.67 ± 0.14 μg/mL) and CRC (6.42 ± 0.19 μg/mL). The ethyl acetate fractions were more potent, with CAE (IC_50_ = 1.63 ± 0.07 μg/mL), followed by CSE (1.93 ± 0.10 μg/mL) and CRE (3.55 ± 0.18 μg/mL). All samples exhibited higher antioxidant activity than the standard BHT (IC_50_ = 8.00 ± 0.24 μg/mL).

In the ORAC assay, all extracts and fractions showed significantly lower EC_50_ values than the standard trolox (EC_50_ = 28.0 ± 14.3 μg/mL), indicating strong peroxyl radical scavenging capacity. Overall, CAE consistently exhibited the strongest antioxidant profile across the three assays and was therefore prioritized for further investigation.

#### 2.1.3. Estimation of the Anti-Elastase, Anti-Collagenase, and Anti-Tyrosinase Activities

The ethyl acetate fraction of *Citrus aurantifolia* (CAE) exhibited pronounced inhibitory activity against elastase, collagenase, and tyrosinase enzymes. These effects were accompanied by relatively low IC_50_ values as shown in Table 1, outperforming both the ethyl acetate fractions of CSE and CRE, as well as their respective hydroalcohol crude extracts (CSC, CRC). Notably, CAE also demonstrated greater enzyme inhibition and lower IC_50_ values in comparison to its own hydroalcohol extract CAC and showed comparable or superior activity relative to the standard reference inhibitors used for each assay. To experimentally validate the molecular modeling insights and assess the contribution of the major bioactive constituent, pure hesperidin was evaluated for its inhibitory potential against elastase and collagenase. As presented in Table 1, hesperidin exhibited remarkable enzyme inhibitory activity, with IC_50_ values of 39.33 ± 1.22 µg/mL (approx. 64.4 µM) against elastase and 212.22 ± 4.33 µg/mL (approx. 347.6 µM) against collagenase.

#### 2.1.4. Correlation Between the Different Values of the Enzyme Inhibitory Assays

**Figure 2:**
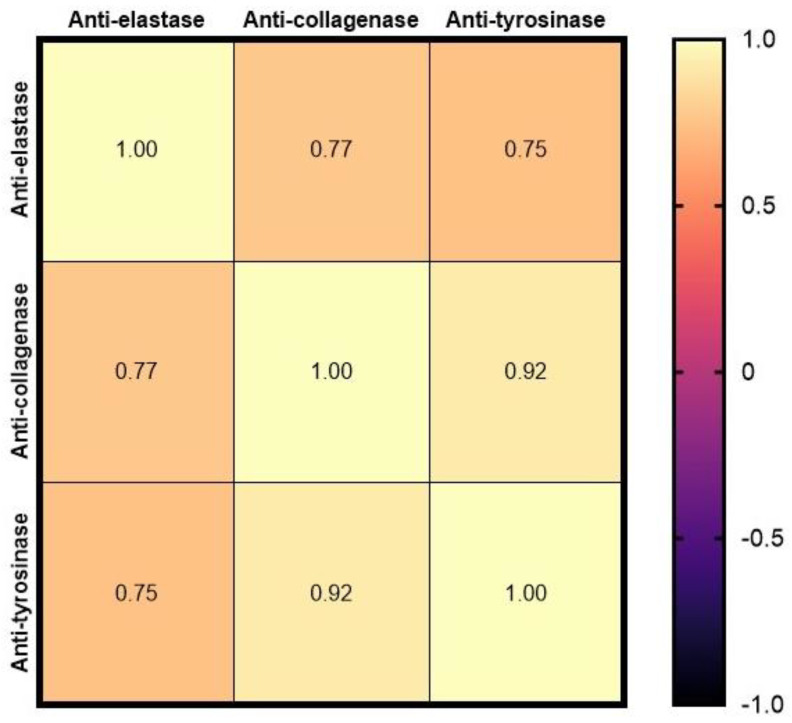
The heatmap displays the Pearson correlation coefficients between the anti-elastase, anti-collagenase and anti-tyrosinase of the different tested samples (CRC, CRE, CAC, CAE, CSC, CSE) (*p* < 0.05) where strong positive correlation for anti-elastase was observed *(r* = 0.77 and *r* = 0.75, respectively), for anti-collagenase (*r* = 0.77 and *r* = 0.92, respectively) and anti-tyrosinase *(r* = 0.75 and *r* = 0.92, respectively). The heatmap’s gradient, visually illustrates the direction and intensity of these relationships.

The Pearson correlation coefficients between the anti-elastase, anti-collagenase and anti-tyrosinase of the different tested samples (CRC, CRE, CAC, CAE, CSC, CSE) were illustrated in the heatmap (Figure 2) and were considered statistically significant at *p* < 0.05 (*p* < 0.05). A strong positive correlation for anti-elastase was observed (*r* = 0.77 and *r* = 0.75 respectively). In addition, strong positive correlation was found for anti-collagenase (*r* = 0.77 and *r* = 0.92, respectively) and anti-tyrosinase (*r* = 0.75 and *r* = 0.92, respectively).

#### 2.1.5. Total Phenolic and Flavonoid Contents

The extraction and fractionation process yielded variable amounts of crude hydroalcohol extracts and their corresponding ethyl acetate fractions among the three *Citrus* species. From 1000 g of dried leaves, the yields were as follows: *C. sinensis* crude extract (CSC, 60.0 g; 6.00% *w*/*w*) and ethyl acetate fraction (CSE, 7.40 g; 0.74% *w*/*w*); *C. aurantifolia* crude extract (CAC, 60.5 g; 6.05% *w*/*w*) and ethyl acetate fraction (CAE, 7.30 g; 0.73% *w*/*w*); and *C. reticulata* crude extract (CRC, 61.75 g; 6.18% *w*/*w*) and ethyl acetate fraction (CRE, 7.10 g; 0.71% *w*/*w*), respectively.

Among the crude extracts, CRC exhibited the highest phenolic content at 68.7 mg gallic acid equivalents (GAE)/g extract, followed by CAC (62.9 mg GAE/g) and CSC (43.9 mg GAE/g). In terms of flavonoid content, CAC showed the highest flavonoid content (48.2 mg rutin equivalents (RE)/g extract), compared with CRC 30.61 mg RE/g extract and CSC 22.1 mg RE/g extract, respectively.

For the ethyl acetate fractions, CRE exhibited the highest phenolic content (116.67 mg GAE/g fraction), followed by CAE (110.82 mg GAE/g) and CSE (105.6 mg GAE/g). Notably, CAE demonstrated the highest flavonoid content among all samples (110.49 mg RE/g fraction), indicating a marked enrichment of flavonoids in this fraction. Together with its superior antioxidant and enzyme inhibitory activities, the high flavonoid content of CAE supported its selection for further phytochemical characterization and nanoformulation.

### 2.2. LC–MS–MS Analysis of Ethyl Acetate Fraction of Citrus aurantifolia CAE

To our knowledge, this is the first report of the polyphenol profile of the ethyl acetate fraction, recognized as the polyphenol-rich fraction of *Citrus aurantifolia* adopting UPLC–PDA–ESI–MS analysis Table 2. A total of fourteen compounds were tentatively identified and compared with literature data, with the majority belonging to flavonoids and their derivatives, alongside a single phenolic acid.

The tentatively identified compounds included flavanone glycosides, such as eriocitrin, naringin, and hesperidin; flavonol derivatives, including rutin, quercetin acetyl hexoside rhamnoside, and quercetin; and C-glycosyl flavones, such as isoorientin, vitexin, vicenin II, and diosmetin derivatives. This profile indicates that CAE is enriched in polyphenolic constituents, particularly flavonoids, supporting its observed antioxidant and enzyme inhibitory activities.

### 2.3. UPLC-PDA Standardization of Ethyl Acetate Fraction of Citrus aurantifolia

**Figure 3:**
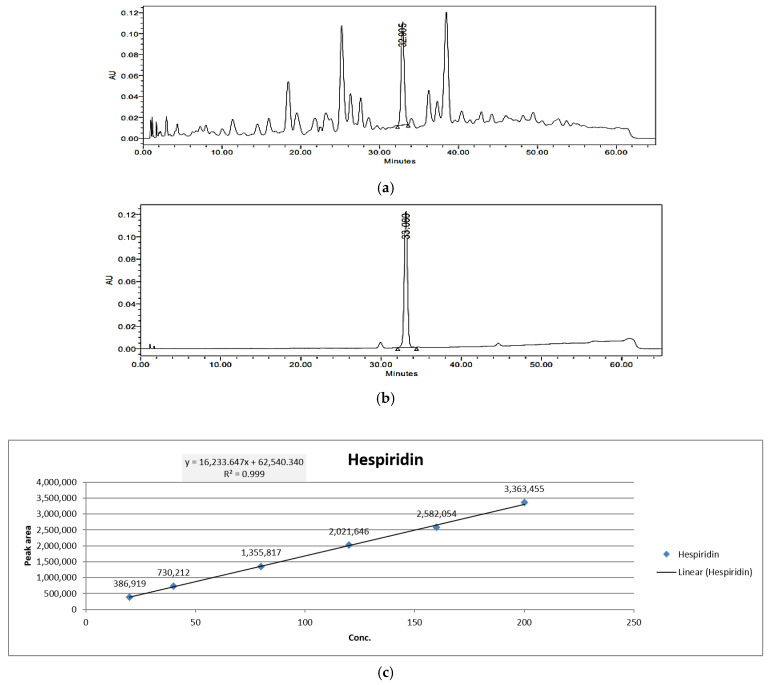
UPLC-PDA standardization of the ethyl acetate fraction of *Citrus aurantifolia* leaves (CAE) using hesperidin as a marker compound. (**a**) UPLC chromatogram of the ethyl acetate fraction; (**b**) UPLC chromatogram authentic hesperidin standard; (**c**) calibration curve of hesperidin.

The ethyl acetate fraction of *Citrus aurantifolia* (CAE) was subjected to analysis by Ultra Performance Liquid Chromatography (UPLC) (Figure 3a). The photodiode array (PDA) chromatogram, recorded at 213 nm, revealed a prominent peak at a retention time (Rt) of 32.905 min, corresponding to hesperidin. Hesperidin was selected because it produced a reproducible and prominent chromatographic peak, was commercially available as an authentic standard, and is a biologically relevant flavonoid associated with antioxidant and skin-protective effects. A calibration curve for hesperidin was generated under the same chromatographic conditions, yielding the equation *y* = 16,233.647*x* + 62,540.340, with a high linearity (R^2^ = 0.999) (Figure 3b). Quantitative analysis indicated that each gram of CAE contained 30.4 ± 0.00139 mg of hesperidin. The validation parameters for the UPLC method, including linearity, LOD, LOQ, recovery, and precision are presented in Table 3.

### 2.4. Evaluation of the Prepared Citrus aurantifolia Ethyl Acetate Fraction-Loaded Span-Based Nanovesicles (CAEnp)

DLS was used to measure the PS, PDI, and ZP of the various formulas. The prepared CAE-loaded Span-based nanovesicles were evaluated for particle size, polydispersity index (PDI), zeta potential (ZP), and entrapment efficiency (EE%), as summarized in Table 4. Incorporation of propylene glycol in F2 improved the physicochemical characteristics of the formulation by reducing particle size and PDI, while increasing the negative zeta potential and entrapment efficiency compared with F1. These findings indicate better size uniformity, colloidal stability, and drug entrapment for F2. Therefore, F2 was selected as the optimized formulation for further investigations and is hereafter referred to as CAEnp.

**Figure 4:**
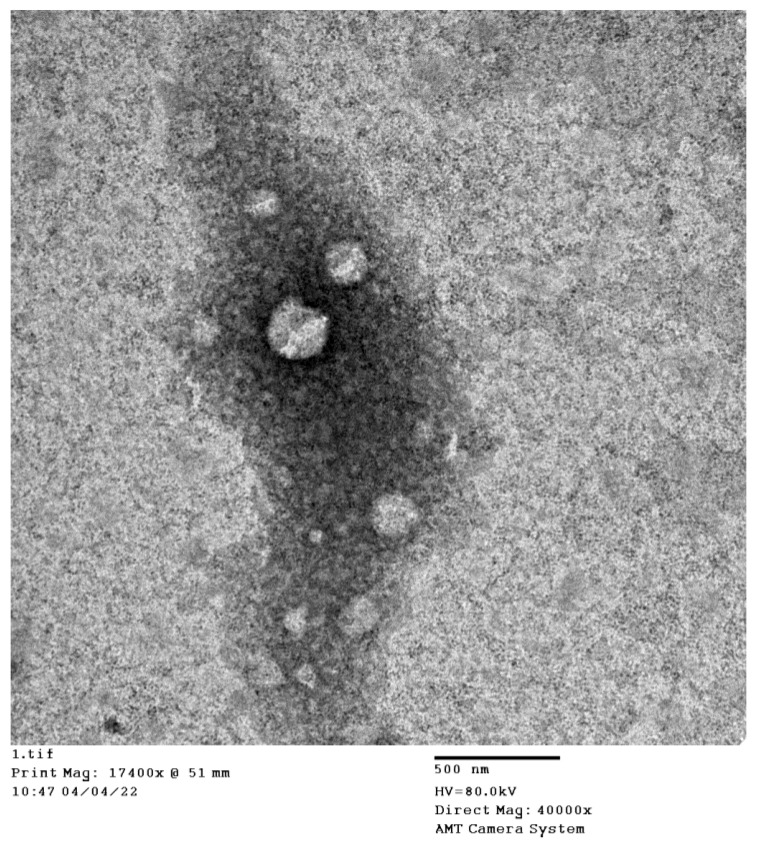
Image of *Citrus aurantifolia* ethyl acetate Fraction-Loaded Span-based nanovesicles (CAEnp).

Morphological investigation of the chosen formula CAEnp using Transmission Electron Microscopy (TEM) (Figure 4) revealed dark, smooth, and spherical nanovesicles.

**Figure 5:**
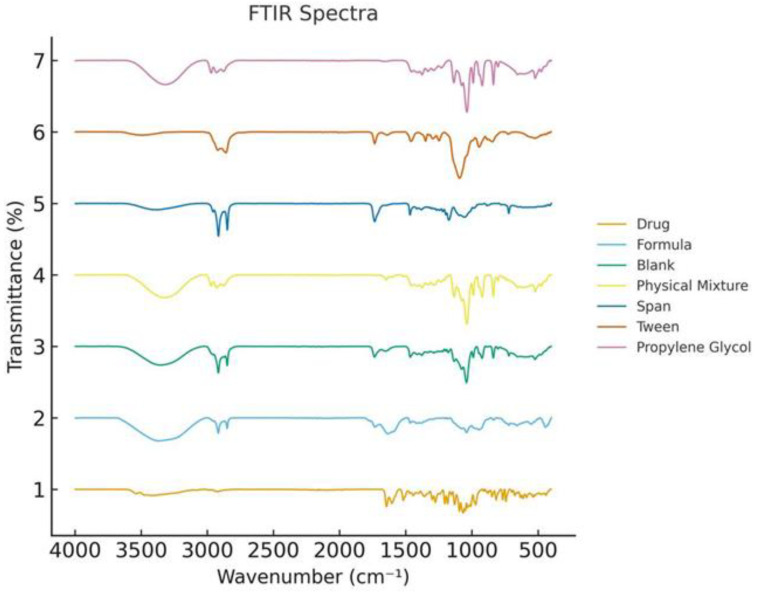
FTIR spectra of the drug (hesperidin), selected formula, blank formula (without drug), physical mixture, and excipients.

As reported in Figure 5, the FTIR spectrum of pure hesperidin exhibited characteristic absorption bands within the range of 3200–3600 cm^−1^, corresponding to O–H stretching vibrations, a distinct band at 1645 cm^−1^ attributable to the C=O stretching of the flavonoid backbone, and additional peaks between 1200 and 1000 cm^−1^ associated with C–O–C and aromatic ring vibrations. These spectral features are consistent with the typical structural characteristics of flavonoid compounds [30]. Span 60 displayed characteristic absorption bands at 3378 cm^−1^ corresponding to aliphatic O–H stretching, at 2916 cm^−1^ and 2849 cm^−1^ associated with aliphatic C–H stretching, and at 1735 cm^−1^ attributed to the C=O ester stretching vibration [31]. Similarly, Tween 80 exhibited prominent peaks at 2921 cm^−1^ and 2858 cm^−1^, which correspond to the asymmetric and symmetric stretching vibrations of methylene (–CH_2_) groups, respectively, in addition to a distinct band at 1735 cm^−1^ related to C=O ester stretching [31]. In addition, the FTIR spectrum of propylene glycol exhibited a broad absorption band around 3317 cm^−1^ corresponding to O–H stretching vibrations, along with peaks in the range of 2970–2876 cm^−1^ attributed to –CH_3_ and –CH_2_ stretching vibrations. The fingerprint region displayed characteristic bands at 1230 cm^−1^, assigned to C–O stretching of secondary alcohols, and at 1136 cm^−1^, corresponding to C–O stretching of primary alcohols [32].

The in vitro release study demonstrated that hesperidin exhibited markedly different release profiles. The free *Citrus aurantifolia* fraction (CAE) showed a rapid hesperidin release, with approximately 90 ± 0.12% of the drug released within the first 4 h, indicating its ability to freely diffuse through the dialysis membrane and confirming that sink conditions were effectively achieved. In contrast, the selected CAEnp nanoformulation exhibited a significantly slower, controlled hesperidin release, with an initial burst release of 13.3 ± 0.9% at 1 h, followed by a sustained release reaching 88.6 ± 3.4% at 24 h. This release pattern, characterized by an initial moderate release followed by a prolonged, gradual phase, suggests a homogeneous entrapment of hesperidin within the nanocarrier system, supporting its potential for sustained drug delivery applications. The prepared gel formulations of the selected formula and the free fraction were yellowish, homogenous, smooth, and had a pH between 5.5 ± 0.16 and 5.9 ± 0.09, which indicates that the formulations are compatible with skin. They are also easily washable with water, have a good spread ability value between 6.1 ± 0.08 and 6.3 ± 0.11 cm. Formulations with high values of spreadability make application easy and increase the surface area available for drug penetration. The gels also show no signs of phase separation and exhibit good consistency throughout the study period.

### 2.5. In Vitro Scratch Wound-Healing Assay of Ethyl Acetate Fraction of Citrus aurantifolia (CAE) and Its Nanoformulaion (CAEnp)

**Figure 6:**
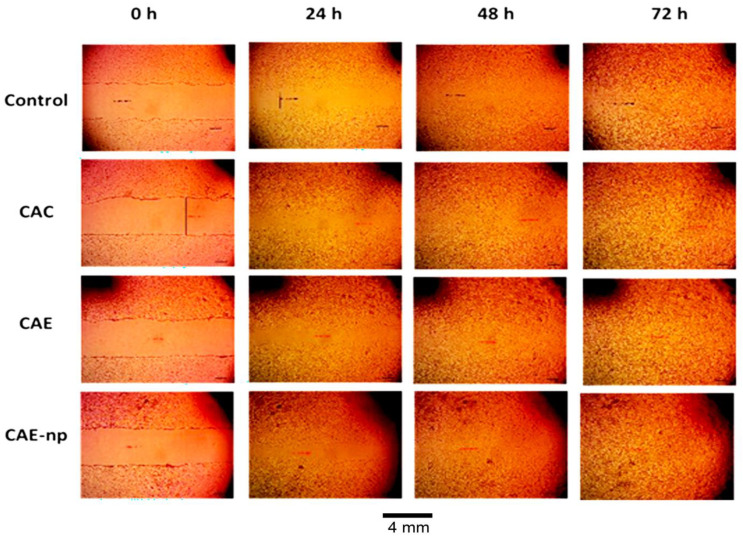
Scratch wound-healing assay in human dermal fibroblasts treated with CAC crude extract, CAE ethyl acetate fraction, and CAEnp nanoformulation. Representative photomicrographs show wound closure at 0, 24, 48, and 72 h after scratch formation. Wound width was measured using MII ImageView software version 3.7, and percentage wound closure was calculated relative to the initial wound width at 0 h. Data are presented as mean ± SD (*n* = 3). Statistical analysis was performed using one-way ANOVA followed by Tukey’s post hoc test. CAE and CAEnp showed significant enhancement of wound closure at 24 h compared with their corresponding controls (*p* < 0.05). Scale bar = 4 mm.

The effects of CAC, CAE, and CAEnp on fibroblast migration were evaluated using an in vitro scratch wound-healing assay (Figure 6). Wound gap closure was monitored at 0, 24, 48, and 72 h and compared with the corresponding untreated controls. All tested preparations enhanced fibroblast migration compared with their respective controls, with CAE and CAEnp showing the most pronounced effects.

At 24 h, CAC treatment reduced the wound width from 3.85 mm to 1.25 mm, corresponding to approximately 67.5% wound closure, compared with 61.7% closure in the untreated control. Although CAC enhanced wound closure, this effect did not reach statistical significance. In contrast, CAE significantly accelerated wound closure at 24 h, achieving approximately 67.5% closure compared with 45.9% in the corresponding control. CAEnp also significantly enhanced wound closure, reaching approximately 67.5% closure at 24 h compared with 50.1% in the control group.

By 48 h, CAE and CAEnp treated cells showed near complete or complete scratch closure; whereas, the corresponding control groups showed delayed closure, particularly in the CAEnp control group. These findings indicate that the polyphenol-rich ethyl acetate fraction and its nanovesicular formulation enhanced fibroblast migratory activity, supporting their potential role in dermal repair-related anti-aging applications.

### 2.6. In Vivo UVA-Induced Photoaging Study

The anti-photoaging effects of CAE and CAEnp were evaluated in a UVA-induced photoaging mouse model by histopathological examination and assessment of oxidative stress- and autophagy-related markers.

#### 2.6.1. Histopathological Evaluation of Ethyl Acetate Fraction of *Citrus aurantifolia* (CAE) and Its Nanoformulation (CAEnp) in UVA-Irradiated Skin

**Figure 7:**
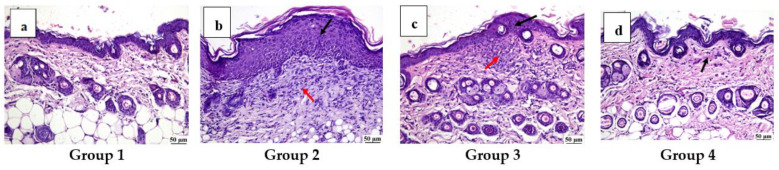
Effect of ethyl acetate fraction of *Citrus aurantifolia* on the histopathological changes in UVA-irradiated skin. Photomicrographs of skin tissue sections from (**a**) the normal control group showing normal structure of epidermis, dermis, and hair follicles, while section from (**b**) UVA-irradiated skin group showing thickened epidermis (black arrow), dermal scaring and inflammation (red arrow). Sections from (**c**) CAE-treated group showing focal epidermal thickening (black arrow) with mild mononuclear cells infiltration (red arrow) and section from (**d**) CAEnp-treated group showing apparently normal skin with mild dermal scaring (black arrow).

Histopathological examination of skin sections from the normal control group showed preserved skin architecture, including normal epidermis, dermis, and hair follicles in Figure 7, where microscopic examination of normal group (Figure 7a) revealed normal histologic structure of skin including epidermis, dermis, and hair follicles. In contrast, the UVA-irradiated group showed marked histopathological alterations, including diffuse epidermal thickening, dysplastic changes, dermal scarring, mononuclear inflammatory cell infiltration, and disruption of skin-associated structures (Figure 7b). The dermis showed scarring with mononuclear inflammatory cell infiltrations and destruction of skin-associated structures including hair follicles. Moderate improvement was detected in the group treated with the formula of CAEnp for four weeks (Figure 7c) following UVA-irradiation. The examined sections revealed focal epidermal thickening and mild dermal scarring, and mild focal dermatitis was also detected. Marked improvement was observed in the group treated with the nanformulation of (CAEnp) for four weeks following UVA-irradiation (Figure 7d), despite the presence of mild dermatitis, sections were apparently normal with only minute areas of dermal scarring and normal hair follicles and skin-associated glands.

#### 2.6.2. Effect of Ethyl Acetate Fraction of *Citrus aurantifolia* (CAE) and Its Nanoformulation (CAEnp) on p62/SQSTM1 in the UVA-Irradiated Skin

**Figure 8:**
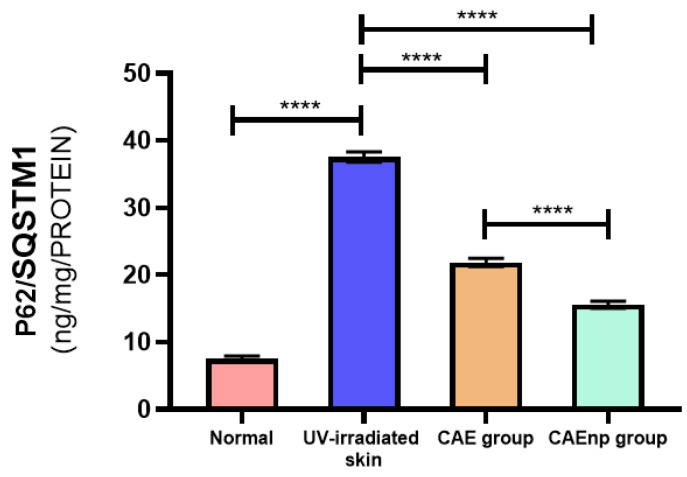
Effect of ethyl acetate fraction of *Citrus aurantifolia* on P62/SQSTM1 content in the UVA- irradiated skin. Data are expressed as mean ± SD, (*n* = 10) and analyzed using one-way ANOVA followed by Tukey’s post hoc test. UVA irradiation significantly increased p62/SQSTM1 levels compared with the normal group (*p* < 0.0001). CAE significantly reduced p62/SQSTM1 levels compared with the UVA group (*p* < 0.0001), while CAEnp produced a further reduction compared with CAE (*p* < 0.0001). **** indicates *p* < 0.0001. P62/SQSTM1: polyubiquitin-binding protein p62/sequestosome 1; CA: Ethyl acetate fraction of *Citrus aurantifolia*; CAEnp: Nanoformulation of ethyl acetate fraction of *Citrus aurantifolia*. Mice were exposed to UVA-radiation (368 nm) for 60 min daily for 6 weeks and then treated topically for 4 weeks.

As reported in Figure 8, UVA-irradiated skin mice showed potential impairment of autophagy and significant accumulation of p62/SQSTM1 content by 399.27% as compared to the normal group (mean ± SD). The group treated with the formula of CAEnp had considerably reduced the P62/SQSTM1 skin accumulation by 41.77% and enhanced the autophagic process as compared to the UVA-irradiated group. Meanwhile, CAEnp produced a stronger reduction by 58.5% and 28.68% compared to UVA-irradiated group and CAE-treated group respectively producing significant autophagy.

#### 2.6.3. Effect of Ethyl Acetate Fraction of *Citrus aurantifolia* (CAE) and Its Nanoformulation (CAEnp) on Keap1 Expression in the UVA-Irradiated Skin

**Figure 9:**
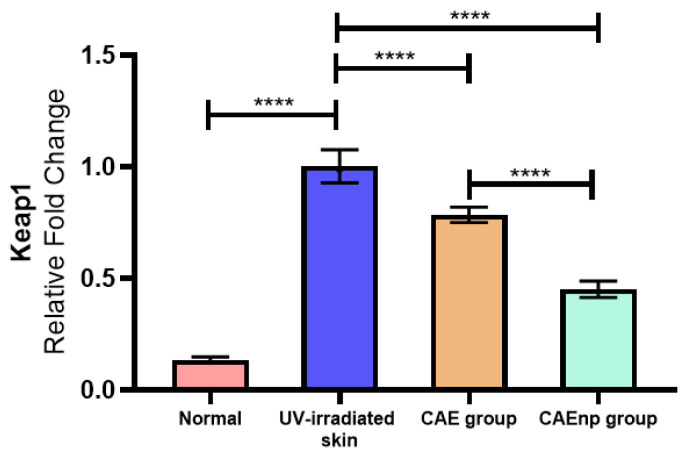
Effect of ethyl acetate fraction of *Citrus aurantifolia* on the relative Keap1 mRNA expression that was determined by quantitative real-time PCR (qRT-PCR), normalized to β-actin, and expressed as fold change relative to the normal control group in the UV-irradiated skin. Data are expressed as mean ± SD, (*n* = 10) and analyzed using one-way ANOVA followed by Tukey’s post hoc test. UVA irradiation significantly increased Keap1 levels compared with the normal group (*p* < 0.0001). CAE significantly reduced Keap1 levels compared with the UVA group (*p* < 0.0001), while CAEnp produced a further reduction compared with CAE (*p* < 0.0001). **** indicates *p* < 0.0001. Keap1: Kelch-like ECH-associated protein 1; CAE and CAEnp were exposed to UVA-radiation (368 nm) for 60 min daily for 6 weeks and then treated topically for 4 weeks.

Keap1 expression was upregulated due to oxidative stress in the UVA-irradiated skin group by 637.5% as compared to the normal group (mean ± SD) as shown in Figure 9. According to the treatment effect in enhancing the antioxidative response, the group treated with ethyl acetate fraction of CAE had reduced skin Keap1 expression significantly by 21.8% when compared to the UV-irradiated group. In addition, the CAEnp downregulated the keap1 expression by 55% and 42.5% compared to UV-irradiated group and the CAE-treated group, respectively.

#### 2.6.4. Effect of Ethyl Acetate Fraction of *Citrus aurantifolia* (CAE) and Its Nanoformulation (CAEnp) on Nrf2 Expression in the UVA-Irradiated Skin

**Figure 10:**
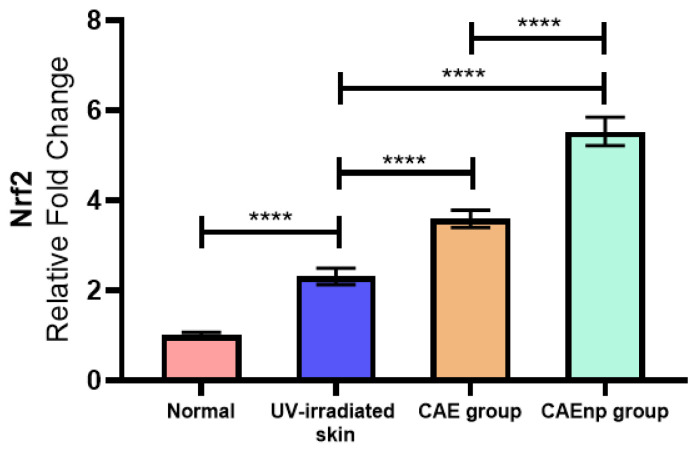
Effect of ethyl acetate fraction of *Citrus aurantifolia* on relative Nrf2 mRNA expression that was determined by quantitative real-time PCR (qRT-PCR), normalized to β-actin, and expressed as fold change relative to the normal control group in the UV-irradiated skin. Data are expressed as mean ± SD, *(n* = 10) and analyzed using one-way ANOVA followed by Tukey’s post hoc test. UVA irradiation significantly reduced Nrf2 levels compared with the normal group (*p* < 0.0001). CAE significantly elevated Nrf2 levels compared with the UVA group (*p* < 0.0001), while CAEnp produced a further elevation compared with CAE (*p* < 0.0001). **** indicates *p* < 0.0001. Nrf2: Nuclear factor erythroid 2-related factor 2; CAE: mice were exposed to UVA-radiation (368 nm) for 60 min daily for 6 weeks and then treated topically for 4 weeks.

As presented in Figure 10, Nrf2 expression fairly increased due to oxidative stress response in UV-irradiated group by 130.94% as compared to the normal group (mean ± SD). In addition, Nrf2 expression elicited a further increase in the group treated with CAE formula by 55.14% when compared to the UVA-irradiated group. Treatment with CAEnp exerted more antioxidant activity proved by a marked increase in the Nrf2 expression by 58.7% and 54.1% when compared to the UV-irradiated group and CAE-treated group, respectively.

#### 2.6.5. Effect of Ethyl Acetate Fraction of *Citrus aurantifolia* (CAE) and Its Nanoformulation (CAEnp) on NQO1 Content in the UVA-Irradiated Skin Mice

**Figure 11:**
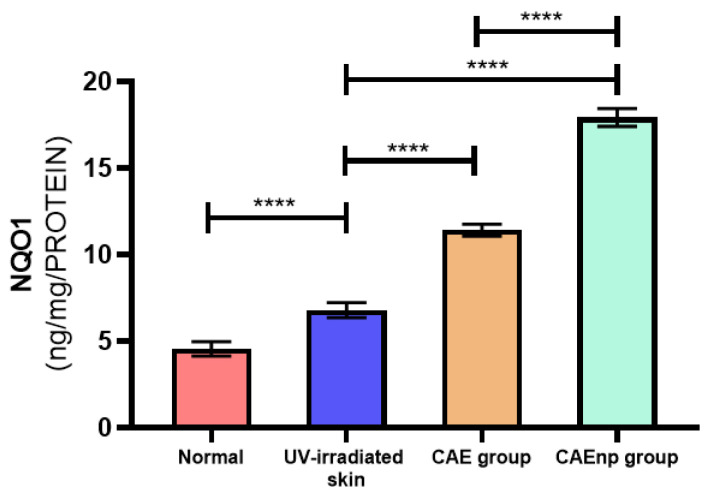
Effect of ethyl acetate fraction of *Citrus aurantifolia* on NQO1 content in the UV-irradiated skin. Effect of *Citrus aurantifolia* on NQO1 skin content. Data are expressed as mean ± SD, (*n* = 10) and analyzed using one-way ANOVA followed by Tukey’s post hoc test. UVA irradiation significantly reduced NQO1 levels compared with the normal group (*p* < 0.0001). CAE significantly elevated NQO1 levels compared with the UVA group (*p* < 0.0001), while CAEnp produced a further elevation compared with CAE (*p* < 0.0001). ). **** indicates *p* < 0.0001. NQO1: NADPH quinone oxidoreductase 1; CAE: ethyl acetate fraction of *Citrus aurantifolia*; CAEnp: nanoformulation of ethyl acetate fraction of *Citrus aurantifolia*. Mice were exposed to UVA-radiation (368 nm) for 60 min daily for 6 weeks and then treated topically for 4 weeks.

The UV-irradiated skin group showed a moderate increase in the skin content of NQO1 by 48.99% when compared to the normal group (Figure 11, mean ± SD). Yet, NQO1 content was considerably increased after treatment with CAE by 68.16% more than the UVA-irradiated skin group. Moreover, the group treated with CAEnp showed obvious elevation by 164% and 57% as compared to the UVA-irradiated skin and CAE-treated group respectively.

### 2.7. Molecular Dynamics

Molecular dynamics (MD) simulations were carried out to investigate the structural and dynamic properties of elastase and collagenase in their apo form, in complex with their native co-crystallized ligand, and in complex with hesperidin (Table 5). The analysis included protein backbone RMSD, radius of gyration (Rg), residue flexibility (RMSF), ligand RMSD, and hydrogen bonding patterns.

**Figure 12:**
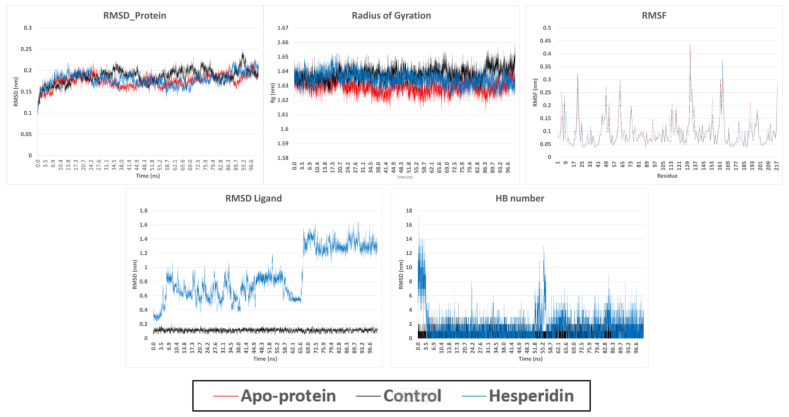
Results of molecular dynamics on elastase.

For elastase (Figure 12), the protein backbone RMSD indicated comparable stability among the three systems. The apo form showed an average RMSD of 0.175 ± 0.015 nm, while the control and hesperidin complexes exhibited slightly higher values of 0.186 ± 0.017 nm and 0.179 ± 0.016 nm, respectively. All these RMSD values are less than 2 Å which indicate reasonable stability of the protein during the production run for all the complexes. Consistent with these findings, the Rg values remained nearly unchanged, with averages of 1.629 ± 0.005 nm for the apo enzyme, 1.638 ± 0.005 nm for the control complex, and 1.635 ± 0.005 nm for the hesperidin complex, indicating preserved compactness. RMSF analysis further confirmed similar residue flexibility across systems, with average fluctuations ranging between 0.095 and 0.097 nm, and no marked changes observed around the active site regions. The RMSF curves for all the three systems were very similar, which indicates similar behavior among residues after binding to both the co-crystalized ligand and hesperidin. The ligand RMSD profile of hesperidin revealed two binding phases. During the first ~66 ns of simulation, hesperidin maintained an average RMSD of 0.669 ± 0.160 nm, after which it transitioned to a new conformation characterized by a higher average RMSD of 1.323 ± 0.090 nm. The second binding mode shows less fluctuation which might indicate a more stable binding. Despite this positional change, the ligand remained well anchored, supported by hydrogen bond analysis, which showed persistent formation of 6–12 bonds, with occasional peaks exceeding 16.

**Figure 13:**
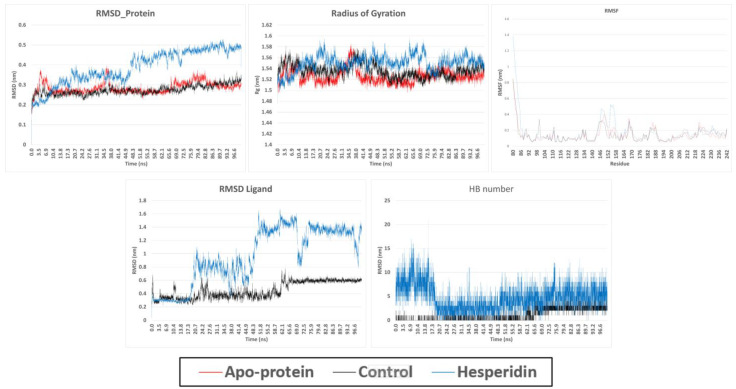
Results of molecular dynamics on collagenase.

In contrast, collagenase exhibited more pronounced structural responses to hesperidin binding (Figure 13). The protein backbone RMSD for the hesperidin complex reached 0.388 ± 0.086 nm, substantially higher than that of the apo enzyme (0.285 ± 0.029 nm) and the control complex (0.274 ± 0.024 nm). This increase was accompanied by a slight expansion in protein compactness, as reflected by Rg values of 1.551 ± 0.015 nm for the hesperidin complex compared to 1.526 ± 0.013 nm and 1.536 ± 0.012 nm for the apo and control systems, respectively. RMSF analysis revealed enhanced flexibility in the hesperidin-bound enzyme, with an average fluctuation of 0.178 ± 0.178 nm, compared to 0.141 ± 0.104 nm in the apo and 0.145 ± 0.100 nm in the control. This might be attributed to the clear increase in RMSF values for residues between 145 and 160 in case of protein–hesperidin complex which are within the loop segments adjacent to the binding site. Analysis of the ligand RMSD showed that hesperidin underwent multiple conformational changes during the trajectory, fluctuating between 0.6 and 1.0 nm during the early 40 ns, and subsequently stabilizing at higher RMSD values exceeding 1.3 nm. Hydrogen bond analysis supported these results, revealing frequent formation of more than eight hydrogen bonds and peaks surpassing 20, demonstrating strong and persistent interactions of hesperidin within the collagenase binding pocket. These results suggest that hesperidin might have better potential for direct binding and inhibition of elastase compared to its interaction with collagenase. It is worth mentioning that biological data of pure hesperidin (Table 1) shows that it has better inhibition of elastase (IC_50_ of around 65 µM) compared to collagenase inhibition (IC_50_ ~345 µM). These preliminary results still require further binding studies between hesperidin and target proteins using other practical techniques such as isothermal calorimetry (ITC) or surface plasmon resonance (SPR).

## 3. Discussion

Extrinsic skin aging is largely driven by environmental stressors such as ultraviolet (UVA) radiation. Long-wavelength UVA-radiation (320–400 nm) penetrates deeply into the dermis and contributes to photoaging, which is characterized by skin thickening, dehydration, wrinkle formation, and pigmentation changes [33,34]. UVA exposure generates reactive oxygen species (ROS) in dermal fibroblasts and keratinocytes, overwhelming antioxidant defenses and disrupting collagen homeostasis [35]. Consequently, extracellular matrix components such as collagen and elastin are degraded by enzymes including matrix metalloproteinase-1 (collagenase) and elastase, leading to loss of skin elasticity and wrinkle formation [36,37]. In parallel, tyrosinase contributes to melanin overproduction and hyperpigmentation [38,39].

In this study, a comparative bioassay-guided approach was used to evaluate hydroalcohol extracts and ethyl acetate fractions from the leaves of three *Citrus* species: *C. sinensis*, *C. aurantifolia*, and *C. reticulata*. Among them, the ethyl acetate fraction of *C. aurantifolia* leaves (CAE) showed the most favorable profile, combining strong antioxidant activity, skin-aging enzyme inhibition, and high flavonoid content. This suggests that ethyl acetate fractionation enriched bioactive constituents and highlights *Citrus* leaves as sustainable sources of polyphenol-rich ingredients for dermal and cosmeceutical applications.

Phytochemical analysis demonstrated significant enrichment of bioactive compounds in the ethyl acetate fraction of *Citrus aurantifolia* (CAE), particularly flavonoids (110.49 mg rutin equivalents/g fraction) and phenolics, consistent with previous reports on *Citrus*-derived polyphenols [40,41]. LC–MS/MS profiling identified fourteen compounds, predominantly flavonoids, with hesperidin as a major marker (30.4 mg/g), in agreement with earlier studies [42,43] and aligns with its recognized antioxidant and skin-protective functions [44]. Beyond hesperidin, CAE also contained naringin, eriocitrin, quercetin, rutin and apigenin derivatives, in agreement with previous *Citrus* phytochemical surveys [43,45,46]. These flavonoids have been linked to skin anti-aging activity through multiple mechanisms. In particular, naturally occurring flavonoids, including quercetin and related flavonols, have been reported to inhibit mammalian collagenase/MMP-1 activity and suppress MMP-1 expression, supporting their possible contribution to the anti-collagenase activity of CAE [47]. Moreover, UVB-induced photoaging is associated with activation of MAPK/AP-1/MMP-1 signaling, leading to collagen degradation; therefore, flavonoid-mediated modulation of oxidative stress and UV induced signaling pathways may indirectly attenuate collagen degrading processes [48]. Rutin has also been reported to increase dermal density, enhance skin elasticity, suppress MMP-1 expression, and reduce wrinkle formation under oxidative stress [49], while naringin improves wound healing and reduces oxidative damage in skin models [50,51]. Therefore, although hesperidin was selected as the major marker compound and showed pronounced anti-elastase and anti-collagenase activities, the overall anti-collagenase effect of CAE should not be attributed to hesperidin alone. The presence of these minor flavonoids may also contribute to collagenase/MMP-1 modulation either through direct enzyme-related effects or through indirect suppression of oxidative stress- and UV-induced collagen-degrading pathways. Together, these findings support the concept that CAE acts as a multifunctional anti-aging fraction, with its biological activity likely resulting from the combined/additive contribution of hesperidin and other bioactive flavonoids.

Consistent with its phytochemical composition, CAE exhibited strong antioxidant activity and significant inhibition of elastase, collagenase, and tyrosinase, aligning with previous findings on *Citrus aurantifolia* extracts [52,53]. Strong positive correlations among antioxidant activity and enzyme inhibition suggest that polyphenol-rich fractions can simultaneously modulate multiple skin-aging pathways. These findings are consistent with previous correlation-based analyses linking polyphenol content to enzyme inhibition and antioxidant capacity [54].

The nanoformulation prepared by ethanol injection using Span 60, Tween 80, and propylene glycol produced nanovesicles with reduced particle size and enhanced flexibility, facilitating improved skin penetration. Formulation CAEnp demonstrated the most favorable physicochemical characteristics, including a smaller PS, narrow size distribution, high EE% of hesperidin, and a sufficiently negative ZP, indicating good colloidal stability. The low PDI value (0.10) obtained by DLS analysis reflects a homogeneous vesicle population in dispersion. Although minor variations in vesicle morphology and apparent size can be observed in the TEM micrograph, such differences are expected due to the distinct nature of the characterization techniques. While DLS provides a population-based measurement of the hydrodynamic diameter of particles in their hydrated state, TEM visualizes a limited number of vesicles following sample preparation and drying. Overall, the TEM findings were in good agreement with the DLS measurements and confirmed the formation of predominantly spherical nano-vesicles.

In addition to the high negative zeta potential, the optimized formulation remained physically stable during a one month temperature variation stability study, showing no signs of phase separation, aggregation, or significant changes in particle size. These findings indicate good short-term physical stability of the formulation. Nevertheless, further long-term stability studies in accordance with ICH guidelines are planned to comprehensively evaluate the formulation under different storage conditions. The inclusion of propylene glycol may have contributed to improved vesicle characteristics and enhanced formulation flexibility, which could support dermal delivery.

FTIR analysis confirmed successful encapsulation of hesperidin, as its characteristic peaks were absent in the nanovesicle spectrum while retained in the physical mixture, indicating no chemical interaction with excipients. Similar FTIR profiles between the optimized and blank formulations further supported system stability. The formulation exhibited sustained drug release, attributed to the rigid bilayer structure of Span 60. Release kinetics followed a diffusion-controlled mechanism, best described by the Korsmeyer–Peppas model (R^2^ = 0.939) and closely aligned with first-order kinetics (R^2^ = 0.935), indicating concentration-dependent release. The calculated release exponent (*n* = 0.840) indicated anomalous (non-Fickian) transport, suggesting that drug release was governed by a combination of diffusion and vesicular matrix relaxation. The sustained, concentration-driven profile is dynamically supported by the incorporation of Tween 80 as an edge activator and propylene glycol as a solubilizer and cosolvent. While Span 60 provides structural integrity, Tween 80 intercalates within the vesicular bilayer, reducing its interfacial tension and imparting high elasticity and ultra-deformability. Concurrently, propylene glycol acts synergistically to increase bi-layer fluidity. Together, these effects may contribute to the controlled and concentration-dependent diffusion of hesperidin from the nanovesicles, thereby supporting the sustained release profile [55,56]. These findings are consistent with a previous report on a similar nanovesicular system [57].

The scratch wound-healing assay showed that CAC, CAE, and CAEnp enhanced fibroblast migration and wound gap closure compared with their corresponding controls. However, statistical significance at 24 h was observed only for CAE and CAEnp, indicating that the polyphenol-enriched ethyl acetate fraction and its nanovesicular formulation exerted stronger pro-migratory effects than the crude extract. The improved performance of CAEnp may be attributed to enhanced delivery and availability of active phytochemicals, supporting the role of nanoencapsulation in improving cellular responses. Since impaired fibroblast function is a hallmark of aged and photoaged skin, the observed enhancement of fibroblast migration may be relevant to dermal repair and anti-aging applications [58,59,60]. Overall, these findings suggest that polyphenol enrichment and nanovesicular formulation may contribute to improved dermal repair-related activity [61].

In vivo, topical application of CAE and its nanovesicular formulation (CAEnp) attenuated UVA-induced epidermal thickening and dysplasia in mice, with CAEnp exhibiting superior effects after four weeks of treatment. These findings are consistent with previous reports describing the wound-healing, antioxidant, and anti-inflammatory effects of *Citrus aurantifolia* extracts and related formulations [62,63] and extend the therapeutic potential to photoaging models.

At the molecular level, CAE activated the Nrf2/ARE antioxidant defense pathway, evident by enhanced Nrf2 and suppression of Keap1 and p62/SQSTM1. Meanwhile, ARE drove transcription of dependent genes that was evident by increased downstream antioxidant enzyme NQO1. This suggests effective mitigation of oxidative stress and regulation of autophagy, essential for maintaining skin cellular homeostasis and delaying aging [64,65,66]. The modulation of p62 may indicate improved autophagy-related turnover. Satsu et al. 2012 [67] showed that Nrf2 activation through the antioxidant response element (ARE) in the NQO1 promoter is directly required for cysteine-induced increase in NQO1 expression. Nrf2’s regulatory function was confirmed when the ARE sequence was mutated, eliminating cysteine-induced promoter activity. Additionally, cysteine treatment enhanced nuclear accumulation of Nrf2 protein while lowering cytosolic Keap1 levels. Moreover, siRNA-mediated silencing of Nrf2 dramatically inhibited NQO1 mRNA expression. Together, our findings demonstrate that Nrf2 activation induces NQO1 at the transcriptional and protein levels, supporting the use of NQO1 protein measurement as a downstream indicator of Nrf2–ARE pathway activity.

Molecular dynamics simulations provided insights into the interaction of hesperidin with elastase and collagenase, revealing stable binding to both enzymes but with distinct structural effects. Elastase maintained structural stability across simulations, indicating binding within a relatively rigid pocket supported by persistent hydrogen bonding. In contrast, collagenase exhibited increased flexibility, particularly in loop regions, suggesting ligand-induced conformational changes. This enhanced flexibility may reflect a mechanism of allosteric modulation, potentially impairing substrate binding and enzymatic activity.

The anti-aging potential of CAE is likely related to its polyphenol-rich phytochemical profile, with hesperidin serving as a major marker compound and one contributor to the observed elastase and collagenase inhibitory activities. However, the overall biological effect of CAE is probably due to the combined action of multiple flavonoids and phenolic constituents rather than hesperidin alone. Collectively, this study demonstrates that comparative bioassay-guided fractionation can identify a polyphenol-rich ethyl acetate fraction from *Citrus aurantifolia* leaves with antioxidant, enzyme-inhibitory, fibroblast pro-migratory, and UVA-protective activities. The nanovesicular formulation enhanced the performance of the selected fraction, supporting the value of advanced delivery systems for improving the topical applicability of plant-derived bioactive fractions. To the best of our knowledge, this is the first integrated study combining comparative *Citrus* leaf screening, phytochemical profiling, UPLC-PDA standardization, Span-based nanovesicular formulation, in vitro and in vivo anti-aging evaluation, and molecular dynamics simulations for a *C. aurantifolia* leaf fraction. Further studies are warranted to confirm long-term stability, skin permeation, clinical safety, and efficacy in human skin models.

## 4. Materials and Methods

### 4.1. Material

Fresh leaves of three *Citrus* species—sweet orange (*Citrus sinensis*), lime (*Citrus aurantifolia*), and mandarin (*Citrus reticulata*)—were collected in November 2021 from the Botanical Garden of the Faculty of Pharmacy, Cairo University (Giza, Egypt). Botanical authentication was carried out by Eng. Therese Labib, botanical consultant at Orman and Qubba Botanical Gardens. A voucher specimen (No. PG005) was deposited in the herbarium of the Faculty of Pharmacy, The British University in Egypt, Cairo.

All solvents used were of analytical grade; whereas, those utilized for UPLC and LC-ESI-HRMS analyses were of HPLC grade. Span 60, propylene glycol, elastase, collagenase, and tyrosinase enzymes, together with the reference standards EDTA, kojic acid, and N-(Methoxysuccinyl)-Ala-Ala-Pro-Val-chloromethyl ketone (purity > 95%), were purchased from Sigma-Aldrich (St. Louis, MO, USA). Ethanol was purchased from Fisher Scientific (Loughborough, UK). All other materials were of analytical grade. In the in vitro scratch wound-healing assay, the human skin fibroblast cell line HSF was obtained from Nawah Scientific Inc. (Mokatam, Cairo, Egypt). Cells were cultured in Fibroblast Growth Kit–Serum-Free medium (ATCC PCS-201-040) supplemented with L-glutamine, hydrocortisone hemisuccinate, HLL supplement (human serum albumin, linoleic acid, and lecithin), recombinant human fibroblast growth factor-β (rhFGF-β), recombinant human epidermal growth factor/transforming growth factor-β1 (rhEGF/TGF-β1), recombinant human insulin, and ascorbic acid. The medium was further supplemented with penicillin (100 U/mL), streptomycin (100 µg/mL), and 10% heat-inactivated fetal bovine serum (FBS). Cells were maintained at 37 °C in a humidified atmosphere containing 5% (*v*/*v*) CO_2_. Wound images were analyzed using MII ImageView software version 3.7.

### 4.2. Plant Extraction and Fractionation

The air-dried leaves of *Citrus sinensis*, *Citrus aurantifolia*, and *Citrus reticulata* (1000 g each) were separately macerated in 70% ethanol (1:5 *w*/*v*) for 72 h at room temperature, with occasional shaking. The extraction was repeated three times using fresh solvent. The extracts were concentrated under reduced pressure at 45 °C to yield the corresponding crude hydroalcohol extracts, which were subsequently suspended in distilled water and successively partitioned with *n*-hexane, dichloromethane, and ethyl acetate. The *n*-hexane and dichloromethane fractions were used solely as a step in the purification and extraction of relatively non-polar components, while the crude extracts and their corresponding ethyl acetate fractions were retained for further phytochemical and biological evaluation [68].

### 4.3. In Vitro Studies

#### 4.3.1. Cytotoxicity Assay in Human Skin Fibroblast

The cytotoxic potential of the hydroalcohol crude extracts (CSC, CAC, and CRC) and their corresponding ethyl acetate fractions (CSE, CAE, and CRE) was assessed in human skin fibroblasts using the sulforhodamine B assay, following the protocol described by Mostafa et al., [69].

#### 4.3.2. In Vitro Antioxidant Assays

The antioxidant activities of the hydro-alcohol crude extracts (CSC, CAC, and CRC) and their respective ethyl acetate fractions (CSE, CAE, and CRE) were evaluated using three complementary in vitro assays. The DPPH radical scavenging assay was conducted following the method described by Mostafa et al., [70,71], with vitamin C serving as the positive control. The oxygen radical absorbance capacity (ORAC) assay was employed to assess the extracts ability to delay fluorescein degradation induced by 2,2′-azobis(2-amidinopropane) dihydrochloride (AAPH), as described by Mostafa et al., [70], using Trolox as a reference standard. Additionally, the β-carotene-linoleic acid bleaching method was applied according to the protocol of Mostafa et al., [69] with butylated hydroxytoluene (BHT) used as the positive control. Antioxidant potency for each extract and fraction was expressed as the concentration required to achieve 50% inhibition (IC_50_).

#### 4.3.3. Evaluation of Skin-Aging-Related Enzyme Inhibition

The anti-aging activities of the hydroalcoholic crude extracts and their ethyl acetate fractions were evaluated using established enzyme inhibition assays in 96-well plates. All experiments were performed in triplicate, with appropriate control and blank wells included for background correction. Percentage inhibition was calculated relative to the untreated enzyme control, and IC_50_ values were determined.

For the elastase assay, human leukocyte elastase was used as the enzyme source. The enzyme solution (1 μg/mL) was mixed with HEPES buffer (pH 7.5) and the tested samples or the reference inhibitor N-(methoxysuccinyl)-Ala-Ala-Pro-Val-chloromethyl ketone. After pre-incubation for 20 min at room temperature, 100 μL of N-methoxysuccinyl-Ala-Ala-Pro-Val-p-nitroanilide substrate (1 mM) was added. The reaction was allowed to proceed for 40 min, and absorbance was measured at 405 nm. Wells containing enzymes and substrates without extracts were used as untreated enzyme control, while sample blanks without enzyme were included to correct for the intrinsic absorbance of the extracts. Samples were tested at concentrations of 25–400 μg/mL [36,69].

For collagenase inhibition, collagenase type I from Clostridium histolyticum (1 mg/mL) was incubated with assay buffer (pH 7.4) and the tested samples or EDTA, which was used as the positive control. After 20 min of incubation at 37 °C, FALGPA was added as the substrate, and the reaction mixture was incubated for a further 1 h at 37 °C. Then, 200 μL of 2% ninhydrin prepared in 200 mM citrate buffer (pH 5.0) was added, and the mixture was heated in a boiling water bath for 5 min. After cooling, 200 μL of 50% isopropanol was added, and absorbance was measured at 540 nm. Untreated enzyme wells were used as controls, and sample blanks without enzyme were included for background correction. All samples were tested at concentrations of 25–400 μg/mL [36,69].

For the tyrosinase assay, mushroom tyrosinase was used as the enzyme source. The enzyme solution (5600 units/mL) was pre-incubated with the tested samples or kojic acid, used as the reference inhibitor, for 15 min at 37 °C. L-DOPA (1 mM) and was then added as the substrate, and dopachrome formation was monitored by measuring absorbance at 475 nm. Wells containing tyrosinase and L-DOPA without extract were used as untreated enzyme control, while sample blanks without enzyme were included to correct for possible color interference from the extracts. Samples were evaluated at concentrations of 75–400 μg/mL [69,72].

### 4.4. Spectrophotometric Determination of Total Phenolic and Flavonoid Content

The total polyphenolic content (TPC) and flavonoid contents (TFC) of the hydro-alcohol crude extracts (CSC, CAC, CRC) and their corresponding ethyl acetate fractions (CSE, CAE, CRE) were quantified using spectrophotometric methods. Total polyphenols were determined by the Folin–Ciocalteu assay and presented as mg equivalent gallic acid/g dry extract. The total flavonoids were assessed using the aluminum chloride colorimetric method and the results are presented as mg equivalent rutin/g dry extract [73]. Based on the initial screening results, CAE was selected for further phytochemical characterization and formulation development.

### 4.5. LC–MS/MS Analysis of the Citrus aurantifolia Ethyl Acetate Fraction

Chromatographic analysis was carried out using a Waters Acquity Xevo TQD system equipped with an Acquity BEH C18 column. The mobile phase consisted of solvent A (0.1% formic acid in water) and solvent B (0.1% formic acid in acetonitrile), delivered at a flow rate of 200 μL/min under gradient conditions as follows: 0–4 min, 15% B; 4–8 min, 20% B; 8–30 min, 55% B; 30–35 min, 90% B; and 35–40 min, returning to 15% B. Samples were prepared in absolute ethanol at a concentration of 1 mg/mL, filtered prior to analysis, and injected at a volume of 10 μL. High-resolution mass detection was conducted using a Xevo triple-quadrupole mass spectrometer coupled with an electrospray ionization source, scanning within an *m*/*z* range of 100–1000 *m*/*z* [74,75].

### 4.6. Standardization of the Citrus aurantifolia Ethyl Acetate Fraction Using Ultra Performance Chromatography (UPLC) Analysis

Standardization of the CAE was conducted using a Thermo Fisher Ultimate 3000 UPLC system (Thermo Fisher Scientific, Waltham, MA, USA) equipped wita PDA–UV–Vis detector. Separation was achieved on a Hypersil GOLD column (250 mm × 4.6 mm, 5 μm particle size). The mobile phase consisted of 0.1% phosphoric acid in water (solvent A) and acetonitrile (solvent B), with a constant flow rate of 0.7 mL/min. The gradient program was applied as follows: 0–7 min (5–15% B), 7–10 min (15% B), 10–22 min (15–35% B), 22–35 min (35–100% B), and 35–40 min (100–5% B). The injection volume was 20 μL, and the column oven temperature was maintained at 30 °C. A calibration curve for hesperidin was prepared over the validated linear concentration range of 20–200 µg/mL. All analyses were performed in triplicate [73]. Hesperidin was selected as the marker compound for standardization because it produced reproducible high-intensity peaks across analytical runs and was commercially available as an authentic standard.

### 4.7. Preparation of Citrus aurantifolia Ethyl Acetate Fraction-Loaded Span-Based Nanovesicles

CAE-loaded Span-based nanovesicles were formulated by using the ethanol injection method [57]. The encapsulation process started by dissolving a precise amount of the extract in a hot (60 °C) Tween^®^ 80 solutions with or without propylene glycol as a penetration enhancer. Afterward, a solution of Span 60 in 2 mL of ethanol was injected dropwise. Each formula had 100 mg of CAE, and the total volume was 10 mL. The resulting hydroalcoholic solution was continuously stirred for 30 min at 800 rpm on a magnetic stirrer to completely evaporate any remaining ethanol and form CAE Span-based nanovesicle dispersions. Subsequently, sonication was performed using an ultrasonic water bath (Crest Ultrasonics Corp., Trenton, NJ, USA) for 5 min to obtain a suitable particle size. The vesicular dispersions were stored at refrigeration temperature for further investigation. The composition of the prepared nanovesicles is displayed in Table 6. Different preliminary formulations with varying Span 60: Tween 80 ratios were initially prepared to identify the most stable nanovesicular system. The 3:1 ratio (300 mg Span 60: 100 mg Tween 80) demonstrated the most favorable particle size, PDI, and zeta potential values; therefore, this composition was selected for further optimization.

### 4.8. Characterization of the Prepared Citrus aurantifolia Ethyl Acetate Fraction-Loaded Span-Based Nanovesicles

#### 4.8.1. Particle Size (PS), Polydispersity Index (PDI), and Zeta Potential (ZP) Measurement

Particle size (PS), polydispersity index (PDI), and zeta potential (ZP) of the prepared formulations were determined using a Malvern Instruments Zetasizer based on dynamic light scattering (DLS) analysis at 25 °C. Prior to measurement, the samples were suitably diluted with deionized water [76].

#### 4.8.2. Entrapment Efficiency Percent (EE%)

Nanovesicles were centrifuged for 2 h at 4 °C and 12,000 rpm, the clear supernatant was collected, and the absorbance was measured to determine the amount of free hesperidin in the CAE-loaded nanovesicles using the UPLC analysis method that was mentioned before. The following equation was employed to calculate the EE% [76].

$$\mathrm{EE}\%=\left(\frac{total\;polyphenol\;rich\;fraction\;concentration-free\;polyphenol\;rich\;fraction\;concentration}{total\;polyphenol\;rich\;fraction\;concentration}\right)\times 100$$

#### 4.8.3. Transmission Electron Microscopy

The morphological appearance of the selected CAE-loaded Span-based nanovesicles formulation was investigated using transmission electron microscopy (TEM) (Jeol JEM1230, Tokyo, Japan) after proper dilution. One drop of the selected formulation was placed on a copper grid and then air-dried at room temperature for 10 min before TEM observation [76].

#### 4.8.4. FTIR Spectroscopy

Fourier Transform Infrared (FTIR) spectroscopy was conducted for the individual components, including hesperidin, Span 60, Tween 80, and propylene glycol, as well as for their physical mixture, the optimized CAEnp formulation, and the corresponding blank (drug-free) formulation. The analyses were performed using an FTIR spectrophotometer (Model 22, Bruker, Coventry, UK) to assess possible interactions between the drug and excipients. Samples were carefully dried, mixed with potassium bromide (KBr), and compressed into translucent disks prior to measurement. Spectra were recorded at 25 °C over the range of 4000–400 cm^−1^ [31].

#### 4.8.5. In Vitro Dissolution Studies

In vitro drug release of free hesperidin in the CAE Fraction from the selected formulation was assessed via the dialysis bag technique and compared with free hesperidin in the CAE [76]. The cellulose dialysis bag was presoaked in distilled water for 12 h. Nanovesicles dispersion equivalent to a known amount of hesperidin in the CAE was exactly weighed, and the same weight of free CAE was resuspended in phosphate-buffered solution (PBS) of pH 7.4 and vortexed for 2 min. The samples were then transferred into the dialysis bags, and both ends of the bags were closed tightly. The bags were immersed in a 50 mL PBS of pH 7.4, which acted as the receptor cell, placed in a shaker water bath at 37 ± 0.5 °C, and shaken at 100 rpm. To ensure sink conditions, 0.1% Tween 80 was added to the phosphate buffer pH 7.4, as reported in previous studies for poorly soluble drugs and structurally related flavonoids. Additionally, aliquots were withdrawn at predetermined intervals (ranging from 1 to 24 h) and immediately replaced with an equal volume of fresh release medium, maintaining constant dissolution volume and preventing saturation [77,78]. Samples were analyzed using the previously mentioned UPLC analysis method. Triplicate samples were measured, and the average concentration was used. In addition, different kinetic models were applied to explore the drug release mechanism. The release rate data were analyzed by fitting them to several models, including zero-order, first-order, Higuchi, and Korsmeyer–Peppas models. The model showing the highest R^2^ value was considered the most appropriate fit.

#### 4.8.6. Formulation of Gels

To improve skin application, the free fraction and the selected nanoformulation were transformed into gels. To form gels with good consistency, the selected formula and the free extract were added to Carbopol gel 1% as the gelling agent and then blended with the use of a magnetic stirrer (Thennolyne Corporation, Dubuque, IA, USA). The prepared gels were examined for various physical characteristics, including phase separation, homogeneity, and clarity. In addition, pH and spreadability were also inspected.

### 4.9. In Vitro Scratch Wound-Healing Assay

For the scratch wound-healing experiment, HSF cells were plated in coated 12-well plates at a density of 2 × 10^5^ cells/well and maintained overnight in DMEM containing 5% FBS until a complete monolayer was achieved. A straight horizontal wound was then created in each well using a sterile pipette tip, and detached cells were removed by washing with phosphate-buffered saline (PBS). Control wells were replenished with fresh culture medium, while treated wells received fresh medium containing the crude extract (CAC), the ethyl acetate fraction (CAE), or the corresponding nanoformulation (CAEnp) at a final concentration of 10 µg/mL. Equal volumes of treatment solutions were added to ensure consistency across experimental groups. Images of the wounded areas were captured at 0, 24, 48, and 72 h using an inverted phase-contrast microscope. Between imaging time points, the plates were incubated at 37 °C in a humidified atmosphere with 5% CO_2_. Image analysis was performed using MII Image View software (version 3.7) [79,80].

Wound width was calculated as the average distance between the opposing edges of the scratch, and wound closure was expressed as the percentage reduction in wound width relative to the initial measurement at 0 h [81]. Data are presented as mean ± standard deviation (SD).

The rate of cell migration (*R_m_*) was calculated using the following equation:

$$R_{m}=\frac{W_{i}-W_{f}}{t}$$

where *W*_i_ represents the average initial wound width, *W_f_* represents the average final wound width, and t is the migration time (hours).

### 4.10. In Vivo Anti-Wrinkle Study of Citrus aurantifolia Ethyl Acetate Fraction

All in vivo experiments were carried out in November 2023 with prior approval from the Ethical Review Committee of the Faculty of Pharmacy, October University for Modern Sciences and Arts (MSA), under the Institutional Animal Care and Use Committee (IACUC) guidelines (approval No. PH86/REC86/2023P, approved on 23 November 2023).

The anti-aging effectiveness of the *Citrus aurantifolia* ethyl acetate fraction and its nanoformulation was assessed using an in vivo model under physiological and histological settings that are not entirely replicable in vitro capturing the relationships between skin structure, cellular metabolism, and the immune response that affect photoaging processes. Specifically, the 3R principles were followed when conducting this investigation particularly Replacement.

#### 4.10.1. Animals and Sample Size

Forty male Swiss albino mice weight 25–30 g were used (purchased from the Egyptian organization for biological products and vaccines). Priori power analysis was conducted using G*Power (version 3.1) for a one-way ANOVA with fixed effects. Assuming a medium-to-large effect size (f = 0.5), α = 0.1, and a power of 0.8, the analysis determined that 40 total observations (*df* = 3, 36; critical F = 2.24) were required, yielding an actual power of 0.816. This calculation confirms that the study design where animals were divided into 4 groups (*n* = 10) provides sufficient statistical sensitivity while adhering to the 3R principles, particularly reduction by ensuring the minimum number of animals necessary for reliable and ethically responsible experimentation. As for Refinement, no animals exhibited signs of discomfort, severe pain, or adverse reactions throughout the experimental period. There was no evidence of infection, significant inflammation, or any other pathological complications. All animals remained healthy, and none required euthanasia or were lost due to treatment-related or ethical concerns.

#### 4.10.2. Experimental Design

The animals were kept for 1 week of acclimatization with free access to food and water. Mice dorsal skin was shaved using hair removal cream then, photoaging was achieved by exposing mice to UV lamp (368 nm) placed on height of 30 cm targeted above the shaved dorsal skin of the mice for 60 min daily for 6 weeks. Forty animals were divided into 4 groups in a stratified random way (*n* = 10). Group 1 was normal mice, who received saline and were not UV-irradiated, group 2 was UV-irradiated (control), group 3 was UV-irradiated then treated with the formulated gel CAE, group 4 was UV-irradiated then treated with the nanoformulated gel of CAE (CAEnp). Treatment (500 mg/kg) [82] was applied to shaved skin three times per week for 4 weeks as reported in [69] and subsequently skin was irradiated with UV. Sample size determined according to G. Power software version 3.1.9.7 (Heinrich Heine University Düsseldorf, Düsseldorf, Germany). At the end of the experimental period, animals were deeply anesthetized by intraperitoneal administration of thiopental sodium (80 mg/kg). Adequate anesthesia was confirmed before euthanasia by the absence of reflex responses. Euthanasia was subsequently performed by cervical dislocation, and death was confirmed by the absence of respiratory movements, cardiac activity, and reflex responses before tissue harvesting. The skin was then excised; one part was stored at −80 °C, and the other part was fixed in 10% neutral buffered formalin. Responsible personnel conducted all measurements in a fully blinded manner with respect to group allocation.

#### 4.10.3. Determination of Skin Content of p62 and NQO1

Using an ELISA-specific kit ((MyBioSource, San Diego, CA, USA), p62 and NAD(P) dehydrogenase-quinone 1 ELISA kits), skin was homogenized in phosphate-buffered saline (PBS, pH 7.4) to measure the contents of skin p62 and NQO1 using manufacturer’s protocol.

#### 4.10.4. Analysis of Keap1 and Nrf2 Gene Expression via qRT-PCR

Relative quantification of gene expression was performed by extraction of RNA from skin cells collected from each experimental group using TRIzol plus RNA purification kit (life technologies, Carlsbad, CA, USA) in accordance with the manufacturer’s protocol. RNA was reverse transcribed using High-Capacity cDNA Reverse Transcription Kit (Applied Biosystems, Foster, CA, USA). Quantification of Keap1 and Nrf2 PCR was carried out using Rotor-Gene Q 5 plex real-time Rotary analyzer (Corbett Life Science, Sydney, NSW, Australia). Quantification of Keap1 RNA and Nrf2 RNA was carried out using PCR fluorescence quantitative diagnostic kit, with SYBR Green PCR Master Mix in triplictes (Applied Biosystems, Foster City, CA, USA). For quantification of Keap1, the primers 5′-ATCCAGAGAGGAATGAGTGGCG-3′ (forward primer) and 5′-TCAACTGGTCCTGCCCATCGTA-3′ (reverse primer) were used. For quantification of Nrf2, primers 5′-CAGCATAGAGCAGGACATGGAG-3′ (forward primer) and 5′-GAACAGCGGTAGTATCAGCCAG-3′ (reverse primer) were used. All were normalized against β-actin 5′-ATCTGGCACCACACCTTC-3′ (forward primer) and 5′-AGCCAGGTCCAGACGCA-3′ (reverse primer). Relative gene expression was calculated using the 2^−ΔΔCt^ method, normalized to β-actin, and expressed as fold change relative to the control group.

#### 4.10.5. Histopathological Examination

Histological evaluation for the skin was done according to the method of O’Keefe et al. [83], and was performed by a blinded pathologist unaware of the treatment allocation.

### 4.11. Statistical Analysis

Data were analyzed with the investigators blinded to group allocation. Results are presented as mean ± standard deviation (SD). IC_50_ values, defined as the concentration producing 50% inhibition, were estimated from linear regression plots of sample concentration versus percentage inhibition. Statistical comparisons were performed using one-way ANOVA followed by Tukey’s post hoc multiple-comparison testing, with six biological replicates (*n* = 6). Differences were considered significant at *p* < 0.05. Pearson correlation analysis was also applied to compare antioxidant activity with enzyme inhibitory effects, with statistical significance set at *p* < 0.05. All analyses were conducted using GraphPad Prism 8 for Windows (GraphPad Software, Inc., La Jolla, CA, USA).

### 4.12. In Silico Molecular Docking Study

Molecular docking was conducted according to our previously described protocol [84] to obtain initial binding conformations for subsequent molecular dynamics simulations. Hesperidin 3D structure was downloaded from Pubchem (www.pubchem.ncbi.nlm.nih.gov, accessed on 25 June 2026). Protein structures were downloaded from protein data bank under pdb codes of 5A8X for elastase (MMP-12) and 5H8X for collagenase (MMP-8). The use of MMP-8 as a representative collagenase was based on the high quality of this specific pdb (resolution of 1.3 Å) and previously reported use [85]. Ligands and proteins were prepared as reported earlier [86]. In summary, water and ions were removed from the protein crystal structures, hydrogens were added and Kollman’s charges were assigned to protein atoms. Docking was done using Autodock Vina [87], with a grid box around the active site (25 Å × 25 Å × 25 Å, centered on cocrystalized ligand) using exhaustiveness of 16. For each target protein, the nine highest-ranked docking poses were evaluated based on binding affinity, and their predicted interactions were examined. The pose showing the most favorable binding energy and a binding orientation comparable to that of the co-crystallized ligand was selected as the starting structure for molecular dynamics simulations.

### 4.13. In Silico Molecular Dynamics Simulations

All-atom molecular dynamics (MD) simulations were conducted using GROMACS version 2020.3 [88], following previously established protocols [89]. Ligand parameters were generated using the SwissParam server [90], while the CHARMM36 all-atom force field [91] was employed to prepare the protein topology. The ligand coordinates derived from molecular docking for hesperidin as reported before [84] and from the crystal structure for the co-crystallized ligand were used to construct the protein–ligand complexes. These complexes were placed in a dodecahedral simulation box and solvated with explicit TIP3P water molecules [92]. Appropriate numbers of Na^+^ or Cl^−^ ions were added to neutralize the system. Energy minimization was carried out using the steepest descent algorithm until the maximum force was below 1000 kJ mol^−1^ nm^−1^. Subsequently, the systems were equilibrated for 1 ns each under NVT then NPT conditions. A 100 ns production run followed. Temperature was maintained at 300 K using the velocity-rescaling (V-rescale) thermostat [93], and pressure was controlled via the Parrinello–Rahman barostat [94]. Bond constraints were applied using the LINCS algorithm [95], and long-range electrostatics were computed using the Particle Mesh Ewald (PME) method [96]. A 2 fs time step was used throughout the simulations, and a 1.2 nm cut-off was applied for van der Waals interactions. Trajectory data from the production phase were processed using *trajconv*, with corrections for periodic boundary conditions (PBC).

## 5. Conclusions

In conclusion, this study demonstrated that *Citrus* leaves, particularly those of *C. aurantifolia*, represent a promising and sustainable source of bioactive compounds for skin anti-aging applications. Through comparative bioassay-guided screening of three commonly cultivated *Citrus* species in Egypt, the ethyl acetate fraction of *C. aurantifolia* leaves was identified as the most active fraction, showing strong antioxidant activity, marked inhibition of skin-aging-related enzymes, and the highest flavonoid content. Phytochemical profiling confirmed its richness in polyphenolic constituents, mainly flavonoids, with hesperidin as a major marker compound. Nanoformulation into Span-based nanovesicles improved its pharmaceutical applicability by providing suitable particle size, good entrapment efficiency, and sustained release. Moreover, CAE and CAEnp enhanced fibroblast migration in vitro and improved UVA-induced histopathological and molecular alterations in vivo, with CAEnp showing superior activity through modulation of oxidative stress markers. The significance of this research lies in transforming underutilized *Citrus* leaf biomass as a value-added natural ingredient for dermal and cosmeceutical applications, supporting sustainability and waste reduction. Further studies, including long-term stability, skin permeation, extended safety assessment, Human Repeat Insult Patch Testing (HRIPT), and clinical validation, are required to support future development into dermatological or cosmetic formulations.

## Figures and Tables

**Table 1:** IC_50_ values of *Citrus* extracts, ethyl acetate fractions and hesperidin compared to the corresponding positive control.

	Anti-Elastase	Anti-Collagenase	Anti-Tyrosinase
	IC_50_	IC_50_	IC_50_
CRC	54.18 a ± 1.12	382.26 b ± 2.8	353.5 c ± 1.03
CRE	53.01 a ± 1.13	314.3 ± 3.08	295.01 c ± 2.12
CAC	48.90 a ± 2.70	300.81 b ± 2.13	280.19 c ± 2.02
CAE	45.5 ± 2.17	253.66 b ± 2.51	180.4 c ± 5.09
CSC	57.2 a ± 2.88	335.34 b ± 2.09	362.5 c ± 2.08
CSE	47.28 ± 1.94	280.71 b ± 3.53	303.03 c ± 3.19
Hesperidin	39.33 a ± 1.22 (64.41 µM)	212.22 b ± 4.33 (347.58 µM)	---
Respective positive control	44.92 ± 1.71	315.12 ± 2.21	321.65 ± 3.41

Data are presented as mean ± SD of three independent experiments each including three technical replicates, (n = 3). (a–c) indicate significant differences from corresponding positive control for anti-elastase, anti-collagenase and anti-tyrosinase activities respectively at *p* < 0.05. Data normality was assessed using the Shapiro–Wilk test prior to one-way ANOVA followed by Tukey’s post hoc test. (CAC, CSC, CRC) represent the hydroalcohol crude extracts and (CAE, CSE, CRE) represent their corresponding ethyl acetate fractions.

**Table 2:** Tentative identification of metabolites detected in the ethyl acetate fraction of *Citrus aurantifolia* using UPLC-PDA-ESI-MS/MS (negative mode).

Peak No.	Retention Time (min)	Identified Compound	Molecular Formula	[M−H]^−^ (*m*/*z*)	Error	Fragment Ions (*m*/*z*)	Class	References
1	9.65	Dihydroferulic acid-hexoside	C_16_H_22_O_9_	357.1207	5.99	295, 223	Phenolic acid derivative	[18]
2	10.22	Isoorientin	C_21_H_20_O_11_	447.0944	3.76	237, 179, 161,135	Flavonoid (C-glycosyl flavone)	[19]
3	10.42	Eriocitrin	C_27_H_32_O_15_	595.1688	4.17	357, 315, 314, 285	Flavonoid (Flavanone glycoside)	[20]
4	10.57	Apigenin di-C arabinoside glucoside	C_26_H_28_O_14_	563.141	1.63	418, 401	Flavonoid (C-glycosyl flavone)	[21]
5	10.64	Vitexin	C_21_H_20_O_10_	431.0999	4.71	365, 179	Flavonoid (C-glycosyl flavone)	[22]
6	10.69	Rutin	C_27_H_30_O_16_	609.1479	3.84	291	Flavonoid (Flavonol glycoside)	[23]
7	11.07	Naringin	C_27_H_32_O_14_	579.1736	3.83	505, 475, 457, 329	Flavonoid (Flavanone glycoside)	[24]
8	11.09	Diosmetin 8-C-glucoside	C_22_H_22_O_11_	461.1108	5.26	623, 461	Flavonoid (C-glycosyl flavone)	[25]
9	11.21	Vicenin II	C_20_H_33_O_20_	593.1548	5.12	191	Flavonoid (C-glycosyl flavone)	[20]
10	11.39	Diosmetin di-C-hexoside	C_28_H_32_O_16_	623.1645	5.28	651, 489, 327	Flavonoid (C-glycosyl flavone)	[26]
11	11.54	Hesperidin	C_28_H_34_O_15_	609.1836	2.72	299	Flavonoid (Flavanone glycoside)	[27]
12	11.78	Diosmin	C_28_H_32_O_15_	607.1688	4.13	328, 313, 298, 28	Flavonoid (Flavone glycoside)	[23]
13	11.86	Quercetin acetyl hexoside rhamnoside	C_29_H_32_O_17_	651.1597	5.52	257	Flavonoid (Flavonol glycoside)	[28]
14	13.15	Quercetin	C_15_H_10_O_7_	301.236	4.32	151, 106	Flavonoid (Flavonol)	[29]

**Table 3:** Validation parameters of UPLC-PDA method.

Validation Parameters	
Linear range	20–200 μg/mL
Correlation coefficient	0.999
Regression equation	*y* = 16,233.647*x* + 62,540.340
Recovery % (mean ± SD)	100.39 ± 1.12
Limit of detection (LOD) (μg mL^−1^)	6.505007778
Limit of quantification (LOQ) (μg mL^−1^)	19.71214478
Precision (RSD%) Intraday precision Interday precision	0.93 1.35

**Table 4:** Physicochemical characterization of CAE-loaded Span-based nanovesicles.

Formulae Code	Particle Size	PDI	ZP	EE%
F1	232.1 ± 2.40 nm	0.25 ± 0.05	−26.5 ± 0.98 mV	72.0 ± 2.7%
F2/CAEnp	184 ± 0.90 nm	0.10 ± 0.01	−30.9 ± 0.75 mV	75.0 ± 1.9%

**Table 5:** Molecular dynamics simulation parameters for elastase and collagenase.

Elastase Enzyme						
	Apo-Protein		Control		Hesperidin	
	Average	STD	Average	STD	Average	STD
RMSD_Protein (nm)	0.175	0.015	0.186	0.017	0.179	0.016
Rg (nm)	1.629	0.005	1.638	0.005	1.635	0.005
RMSF (nm)	0.096	0.058	0.097	0.059	0.095	0.055
RMSD_Ligand (nm)	NA	NA	0.113	0.021	0.888	0.340
HBN	NA	NA	0.665033	NA	1.50065	NA
Collagenase Enzyme						
RMSD_Protein (nm)	0.285	0.029	0.274	0.024	0.388	0.086
Rg (nm)	1.526	0.013	1.536	0.012	1.551	0.015
RMSF (nm)	0.141	0.104	0.145	0.100	0.178	0.178
RMSD_Ligand (nm)	NA	NA	0.452	0.123	0.965	0.426
HBN	NA	NA	1.030	NA	4.652	NA

Summary of molecular dynamics simulation parameters for elastase and collagenase in apo form, native ligand-bound (control), and hesperidin-bound states. The reported values represent the average over 100 ns trajectories, with standard deviations (STD) where applicable. RMSD: root mean square deviation of protein backbone (nm); Rg: radius of gyration (nm); RMSF: root mean square fluctuation of residues (nm); RMSD Ligand: ligand RMSD with respect to its initial position (nm); HBN: average number of hydrogen bonds between ligand and protein. NA: not applicable (e.g., no ligand in apo form).

**Table 6:** Composition of the prepared Span-based nanovesicles loaded with *Citrus aurantifolia* ethyl acetate fraction.

Formulae Code	Span 60: Tween 80 Ratio	Propylene Glycol (%)
F1	3:1	0
F2	3:1	1

## Data Availability

The data that support the findings of this study are openly available.

## Abbreviations

AAPH	2,2′-Azobis(2-amidinopropane) Dihydrochloride (free radical generator)
ARE	Antioxidant Response Element
BHT	Butylated Hydroxytoluene (antioxidant standard)
CAE	*Citrus aurantifolia* ethyl acetate fraction
CAC	*Citrus aurantifolia* crude extract
CSE	*Citrus sinensis* ethyl acetate fraction
CSC	*Citrus sinensis* crude extract
CRE	*Citrus reticulata* ethyl acetate fraction
CRC	*Citrus reticulata* crude extract
CAEnp	Nanovesicular formulation of *Citrus aurantifolia* ethyl acetate fraction
Keap1	Kelch-Like ECH-Associated Protein 1
MD	Molecular Dynamics
MMP-1	Matrix Metalloproteinase-1 (collagenase)
NQO1	NAD(P)H Quinone Oxidoreductase 1
Nrf2	Nuclear Factor Erythroid 2-Related Factor 2
ORAC	Oxygen Radical Absorbance Capacity
P62/SQSTM1	Sequestosome 1 (autophagy marker protein)
PBS	Phosphate-Buffered Saline
PCA	Principal Component Analysis
PG	Propylene Glycol
RE	Rutin Equivalents
Rg	Radius of Gyration
RMSD	Root Mean Square Deviation
RMSF	Root Mean Square Fluctuation
SRB-U	Sulforhodamine B (Uptake) cytotoxicity assay
